# Supplementary material for: Surface hydrophilicity promotes bacterial twitching motility
Source: mSphere. 2024 Aug 28;9(9):e00390-24. doi: 10.1128/msphere.00390-24 (PMC11423576; doi:10.1128/msphere.00390-24)
Supplement: Supplemental material — Tables S1 to S3; Fig. S1 to S3. [file msphere.00390-24-s0001.pdf]

Supplemental Materials:

Table S1. Ingredients of LB (Fisher) and MacConkey (OXID) agar media for twitching.

**MacConkey (MC) agar***	**Luria-Bertani (LB) agar***
1.2% Agar	1.2% Agar
0.5% Sodium Chloride	0.5% Sodium Chloride
0.075% Neutral Red	0.5% Yeast Extract
2% Peptone#	1% Tryptone
1% Lactose#	
0.5% Bile Salts#	

*Ingredient concentrations are in % (w/v).
Ingredients highlighted in blue signify key differences in media composition.

Table S2. Properties of detergents.

Detergent	MaxNIC (µg/mL)*	CMC (mM)**	Type**
**SDS**	750	7-10	Anionic
**Triton-X100**	75	0.23	Non-ionic
**Triton-X114**	75	0.2	Non-ionic

* The maximum non-inhibitory concentration (MaxNIC) of each detergent against *P. aeruginosa* PAO1 was determined experimentally in liquid growth media in the presence of detergent at different concentrations (data not shown).

** CMC (critical micelle concentration) and types of detergent were from the reference data from Sigma-Aldrich (Bhairi et al., 2017).

Table S3. Antimicrobial susceptibility testing of *P. aeruginosa* strain PAO1.

Antibiotic	MaxNIC (ng/ml)*	Target
Ampicillin	313	Cell wall synthesis
Ciprofloxacin	31	DNA gyrase
Gentamicin	31	30S ribosomal rRNA
Polymyxin B	313	Outer membrane

* The maximum non-inhibitory concentration (MaxNIC) of each antibiotic against *P. aeruginosa* PAO1 was determined experimentally by liquid growth media in the presence of antibiotic at different concentrations (data not shown).

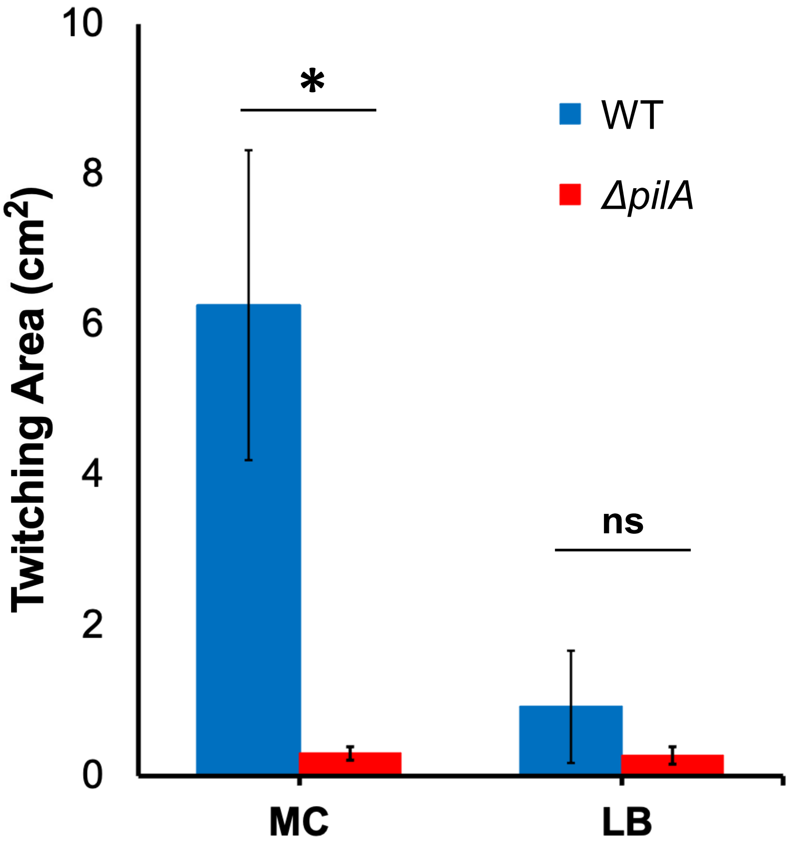

Figure S1. Twitching motility of *Acinetobacter nosocomialis M2* on MacConkey and LB agar. Twitching motility of wildtype (WT) and an isogenic *pilA* mutant (*ΔpilA*) of *A. nosocomialis* strain M2 was analyzed and presented as in Figure 1A. Data presented is from the same experiment as Figure 1A; three biological experiments each performed in triplicate. An asterisk (*) specifies that the twitching area values with bile salts for the indicated strains are statistically different ($P<0.05$) from those without bile salts, by the Student's T-test.

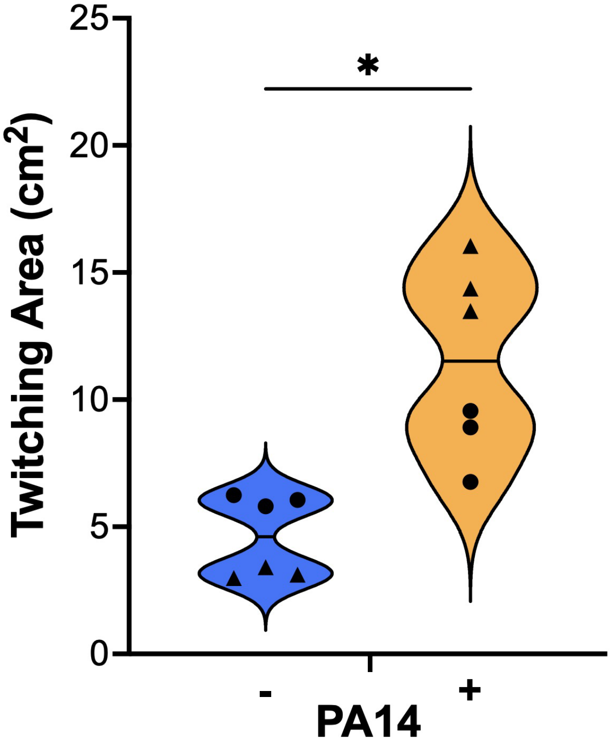

Figure S2. Twitching motility of *Pseudomonas aeruginosa* PA14 in the presence of bile salts. Twitching motility was analyzed and presented as in Figure 1B. Data presented is from two biological experiments each performed in triplicate. An asterisk (*) specifies that the twitching area values with bile salts for the indicated strains are statistically different (P<0.05) from those without bile salts.

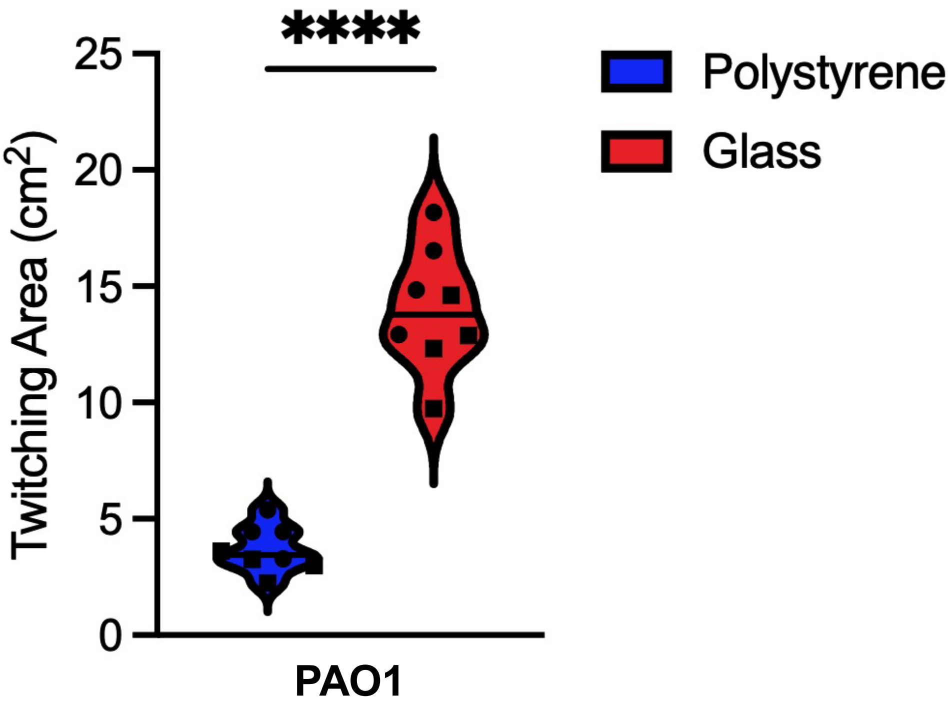

Figure S3. Glass surfaces enhance the twitching motility of *Pseudomonas aeruginosa*. Twitching motility of *P. aeruginosa* PAO1 were analyzed on polystyrene or borosilicate glass Petri dishes in Luria-Bertani (LB) agar, as in Figure 1B. Data presented is from two biological experiments each performed in quadruplicate. Quadruple asterisks (****) specifies that the twitching area values with bile salts for the indicated strains are statistically different ($P<0.0001$) from those without bile salts, by the Student's T-test.

Supplemental References:

Bhairi, S.M., Mohan, C., Ibryamova, S., LaFavor, T. *A guide to the properties and uses of detergents in biological systems*. 2017; Available from: https://www.sigmaaldrich.com/deepweb/assets/sigmaaldrich/marketing/global/documents/186/820/detergents-guide-ms.pdf.
